# Whiteness and (anti-) racism in physical education considering teachers’ and physical education teacher education students’ perspectives: a systematic review

**DOI:** 10.3389/fspor.2026.1903712

**Published:** 2026-10-14

**Authors:** Lucas Abel, Annette Chidinma Galle, Ivie Ojo, Tobias Vogt

**Affiliations:** Institute of Professional Sport Education and Sport Qualifications, German Sport University Cologne, Cologne, Germany

**Keywords:** critical pedagogy, discrimination, marginalization, social justice, teacher education

## Abstract

**Introduction:**

While Physical Education (PE) can combat racism, it also risks perpetuating racist attitudes and behaviors. The aim of this study is to analyze international literature on racism and anti-racism in the context of PE through teachers' and physical education teacher students' (PETE) perspectives.

**Methods:**

Guided by PRISMA, the systematic review follows a four-step process: (1) searching nine databases using 70 keyword combinations, (2) applying five inclusion criteria, (3) assessing study quality, and (4) conducting template and reflexive thematic analyses. Of the 3,346 publications identified, 23 were included in the analysis.

**Findings:**

Four themes were constructed through thematic analysis: (i) “How racism is understood”, (ii) “Perspectives of PE Teachers and PETE Students”, (iii) “Unveiling Whiteness”, and (iv) “Addressing Gaps in Anti-Racist training.” Analysis reveals that racism in PE is a multifaceted phenomenon shaped by local contexts, with whiteness operating as an invisible norm. Teachers are confronted with racism both through their own experiences and through witnessing students being subjected to racism. Some educators advocate for anti-racism training to address biases and systemic inequities, yet current PE curricula lack sufficient anti-racist content.

## 1 Introduction

Discrimination and racism remain prevalent in Germany and across Europe in various domains (1). Racialized groups still face unequal treatment (2). This highlights the need for tolerance and inclusion to uphold democracy (3). As early as 1935, Dr. E.B. Henderson saw physical education (PE) as a tool for fostering tolerance (4). In their article, Clark et al. (4) describe Henderson as a catalyst for this. PE is well-positioned to foster tolerance and social cohesion. However, through its country-specific monitoring activities, the European Commission against Racism and Intolerance (ECRI) has also underscored how racism continues to manifest in sports and is often exacerbated by the tendency to downplay instances of racial discrimination (5). Within the context of sports, PE warrants particular attention due to its pedagogical role. Thus, PE can either critically address or unintentionally reinforce racism (6). In its pedagogical function, PE grants access to sports culture and can inspire long-term engagement, influencing personal development (7). From a Critical Race Theory (CRT) perspective, PE may reproduce dominant ideologies or serve as a space of resistance (8). However, this potential is not guaranteed. PE can also be colorblind and male-dominated (8, 9). This underlines the need to challenge normative structures to unlock PE's anti-racist potential.

Racism is a global but locally variable phenomenon (10), and theories reflect this diversity (11). While multiple theoretical frameworks complicate its understanding, they simultaneously enrich the discourse.

In light of the aforementioned complexity, teachers' perspectives on anti-racism research within education are crucial, as they provide first-hand insights into classroom dynamics, student interactions, and institutional policies. Positioned on the frontline, teachers witness instances of racism and its impact on students' academic and social development (12). Their experiences are crucial in identifying systemic issues, assessing current anti-racism strategies, and crafting practical interventions. Teachers' insights highlight power dynamics in education, exposing how authority can sustain or challenge racism. These perspectives ground anti-racism efforts in real educational contexts, thereby enhancing their relevance and impact (13).

Despite the recognized challenges and opportunities within the field, to the best of our knowledge, no comprehensive review of the existing research has yet been undertaken. A similar review focusing on students' perspectives has previously been published by the authors of this article. However, the datasets used in the two publications differ fundamentally. This study aimed to investigate the availability and quality of both national and international publications on racist experiences and anti-racist practices in PE from teachers' perspectives. Additionally, we aimed to assess whether actionable recommendations for enhancing anti-racism in PE can be derived from the existing literature.

To answer the research question: “What does the existing literature reveal about the experiences and perceptions of racism among PE teachers and Physical Education Teacher Education (PETE) students, and what are the identified implications for anti-racist practices and policies in PETE programs?” a systematic review was conducted, followed by a thematic analysis.

## 2 Theoretical framework

Racism is a global issue with local characteristics (10). It comprises various types such as individual racism (experiences within the personal sphere) and institutional/structural racism (racist discrimination stemming from established institutions or at the structural level) (14).

CRT, developed by Black scholars in the late 20th century, challenges systemic racial inequality and white Supremacy while emphasizing race as a social construct. Furthermore, CRT emphasizes intersectionality—how overlapping marginalized identities shape oppression (15). Adapting the overarching theoretical framework of CRT—initially developed in the U.S.—proves insightful for comprehending the mechanisms of racism in a European context, as suggested by Gillborn (16). The successful application of CRT in education highlights the perpetuation of power relations in social systems through the construction of race and racism in schools (17). In PETE, scholars strive to challenge white Supremacy and racism using CRT principles (4, 18–20). Derived from CRT, Critical Whiteness Studies (CWS) critiques whiteness as an unmarked norm and its influence on institutions (21). Aligned with CRT, CWS acts as a tool of resistance, challenging the presumed neutrality of whiteness and exposing its role in educational inequity (21). In PE, CRT and CWS provide a lens for examining how racialized and white normative assumptions can become embedded in embodied practices, performance evaluation, and organizational routines such as grouping and team selection (20, 22). These perspectives shift attention from individual prejudice to the ways pedagogical and institutional practices may reproduce or challenge racialized hierarchies. The study of racism in Germany—as shaped by its history—extends beyond anti-Black racism. Against this background, the German Center for Integration and Migration Research identifies six focus groups in the National Discrimination and Racism Monitor: Black persons, Muslims, Asians, Sinti and Roma, Jews, and Eastern Europeans (23). We acknowledge and take this diverse understanding of racism into account.

## 3 Methods

### 3.1 Authors' positionality and perspective

In accordance with other research in the field of CRT in PE, we acknowledge the importance of outlining our positionality. The first and last authors identify as white men, both holding a teaching position at the university, while the last author also holds a professorship at the corresponding institute. The second and third authors identify as Black women, working as research assistants at the institute. All authors acknowledged work- and race-related power dynamics and reflected on how these influenced the collaboration. The role of the third author was to complement the work of all authors with practical, in-field insights. This perspective is essential in CRT, where PoC experience is valued (24). In accordance with CRT, the Black female authors challenged the perspectives of their white colleagues to unveil potential biases. All authors are German citizens.

### 3.2 Search strategy

The search was conducted in March 2026, across nine databases, encompassing both German and international repositories: Surf, FIS, BASE, Web of Science, Scopus, The Lens, African Journals OnLine, DOAJ, and ERIC. A total of 70 keyword combinations were used, each consisting of two exposures (Table 1). The operator “AND” linked the two exposures, while “OR” connected the keyword combinations.

**Table 1:** Keyword combinations.

Exposure 1	Exposure 2
“physical education,” “school sport,” “physical education teacher training,” “physical education workshop,” “physical education advanced training”	“racism,” “racism research,” “racist experience,” “racism critique,” “anti-racism,” “critical whiteness,” “critical race theory”

Search terms (in English and German) were applied across all databases without date restrictions. The following selection criteria, outlined according to the Preferred Reporting Items for Systematic Reviews and Meta-Analyses (PRISMA standard), were used to screen and include or exclude—if applicable—potentially relevant studies (25): (a) publication in an international, peer-reviewed journal; (b) publication in English or German; (c) study population involving students, PE teachers, or PETE students; (d) focus on (anti-) racism in PE; and (e) inclusion of all identified forms of racism as defined by the author(s). Studies were selected by reviewing titles and abstracts identified during the search. Subsequently, two researchers independently examined the full texts. Discrepancies were addressed through discussion. This process is outlined in Figure 1. The database search identified 3,346 records, of which 46 publications met the inclusion criteria. Screening the reference lists yielded 10 additional publications, resulting in 56 eligible publications overall. These covered student, teacher, and researcher perspectives; the present review focuses on the 23 publications addressing PE teachers and PETE students.

**Figure 1:**
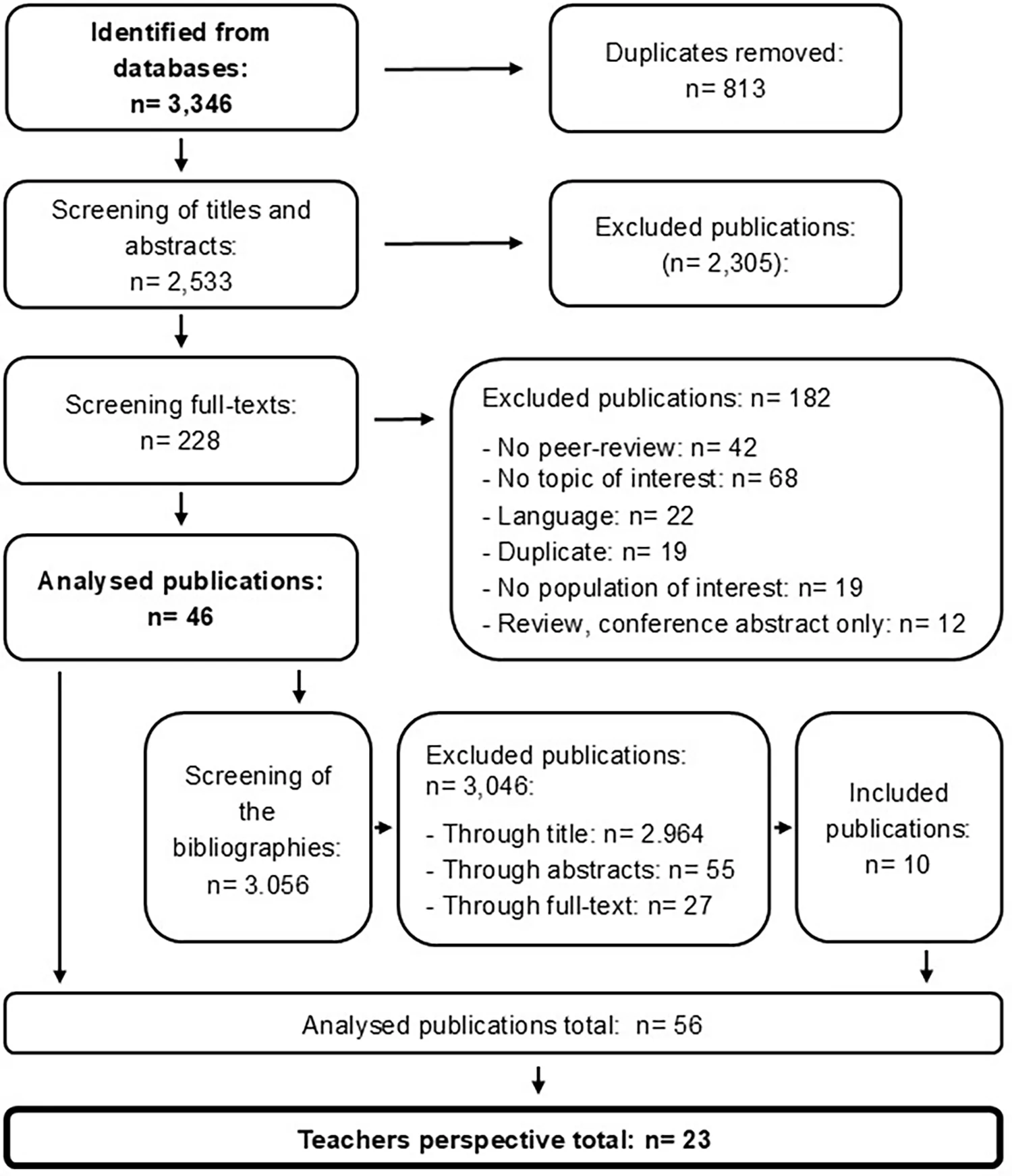
Flow chart.

### 3.3 Assessment of study quality

The quality of the qualitative publications was assessed using the Critical Appraisal Skills Programme (26). The Mixed Methods Appraisal Tool (MMAT) was used for publications utilizing quantitative and mixed-methods approaches (27). Checklists were binary (“yes”/“no”). While the checklists used in this investigation inherently lacked a built-in rating system, the quality rating used in this study was adapted from Silva et al. (28). The following ratings were applied: Qualitative publications were rated as low quality (LQ) if 0–3 questions were answered with “yes”, medium quality (MQ) if 4–6 questions were answered with “yes” and high quality (HQ) if 7–10 questions were answered with “yes”. For quantitative publications, a study was rated as LQ if 0–3 questions were answered with “yes”, MQ if 4–5 questions were answered with “yes,” and HQ if 6–7 questions were answered with “yes”. Regarding mixed method approaches, a study was rated as LQ if 0–6 questions were answered with “yes”, MQ if 7–12 questions were answered with “yes”, and HQ if 13–17 questions were answered with “yes”. Two authors independently assessed study quality and discrepancies in their evaluations were reconciled through a consensus meeting. As all discrepancies were resolved by consensus, involvement of a third reviewer was not required. The theoretical publications listed were not subjected to quality assessment.

### 3.4 Data analysis

For each publication, the following attributes were compiled and summarized: title, author(s), country, year of publication, objectives, methodology, and quality assessment (Table 2). Thematic analysis (TA) was used to examine all 23 publications focusing on PE teachers and PETE students. TA was chosen for its flexibility in identifying patterns across heterogeneous literature at both semantic and latent levels, while allowing the researchers' positionalities to be explicitly considered in the interpretive process. The four themes were not defined *a priori* but were constructed iteratively through coding and repeated engagement with the dataset. During this process, we observed that the first two themes were based primarily on semantic codes that summarized and described the data, resulting in descriptive topic-summary themes. Themes three and four drew more strongly on latent codes and interpreted the data within a broader social and political context, resulting in more interpretive shared-meaning themes. In consequence, we applied template analysis to the first two themes as a form of codebook TA. Additionally, we adapted the analysis process by generating a codebook within the process of reviewing themes (29). Themes three and four were created through reflexive TA following additional recommendations from Braun and Clarke (30). As authors, we actively shaped the themes, influenced by our perspectives and our long-term goal of developing a teacher-training module (31). CRT served as an analytical lens throughout this iterative process, guiding how we interpreted and synthesized the literature. Principles including intersectionality, counter-storytelling and the critique of whiteness shaped both our TA and our interpretation of findings. Constant refinement and expansion of the template occurred during data familiarization, allowing for a conceptual understanding of the dataset.

**Table 2:** Results of systematic review .

Reference number	Title	Authors	Year	Objectives	Methodology	QR
32	Promoting Equity Awareness in the Preparation of Physical Education Students	Kay M. Williamson and Jacqueline A. Williams	1990	The article aims to evaluate and report on a university class with the aim to prepare future physical education teachers by promoting awareness and addressing issues of inequality, discrimination and biases in PE.	Conceptual/theoretical	
33	“Just open your eyes a bit more”: the methodological challenges of researching black and minority ethnic students” experiences of physical education teacher education	Anne Flintoff and Louisa Webb	2012	“In this paper we discuss some of the challenges of centralizing race and ethnicity in Physical Education (PE) research, through reflecting on the design and implementation of a study exploring black and minority ethnic (BME) students experiences of their teacher education.”	Conceptual/methodological	
34	The Wages of Whiteness: Confronting the Nature of Ivory Tower Racism and the Implications for Physical Education	Delia D. Douglas and Joannie M. Halas	2013	“we present the results from two interconnected studies designed to make transparent: (1) the demographic character of faculties of Kinesiology and Physical Education across Canada, as well as (2) the delivery of content related to race, diversity and whiteness within the curriculum”	Mixed methods	HQ
35	Tales from the playing field: black and minority ethnic student´s experiences of physical education teacher education	Anne Flintoff	2014	“This article presents findings from recent research exploring black and minority ethnic (BME) students experiences of Physical Education teacher education (PETE) in England.”	Qualitative	HQ
36	Playing the “Race” card? Black and minority ethnic students’ experiences of physical education teacher education	Anne Flintoff	2015	“This paper reports on a study that explored black and minority ethnic (BME) students’ experiences of physical education teacher education (PETE) in England.”	Qualitative	HQ
37	On racism and prejudice: exploring post-critical possibilities for service-learning within physical education teacher education	Judy Bruce	2015	“Through this self-study, I wanted to reflect upon my own experiences of teaching using a post-critical pedagogy, and this included a critical reflection of my responses to PETE students beliefs, attitudes and recollections of experiences.”	Qualitative	HQ
38	Disrupting racialization: considering critical leadership in the field of physical education	Katie Fitzpatrick and Lorri J. Santamaría	2015	“Drawing on the theoretical foundations of transformational leadership, critical pedagogy, and critical race theory, we draw links between the field of PE and applied critical leadership.”	Conceptual/theoretical	
39	Stereotypes and prejudices in pre-service physical education teachers towards immigrants	Pastor-Vicedo, J.C., Contreras- Jordán, O.R., Gil-Madrona, P. and Cuevas-Campos, R.	2016	“The purpose of this study was to find out which stereotypes prevail in future Physical Education (PE) teachers when asked about immigrant population and to see if within that stereotype, there are subtle or blatant racist attitudes, as well as egalitarian attitudes.”	Quantitative	HQ
40	African American Teacher Candidates Experiences in Teaching Secondary Physical Education	Takahiro Sato and Samuel Russell Hodge	2017	“The purpose of this study was to describe and explain the teaching experiences of African American physical education teacher candidates in secondary physical education programs at urban schools.”	Qualitative	HQ
41	Conceptualizing social justice and sociocultural issues within physical education teacher education: international perspectives	Joanne Hill, Rod Philpot, Jennifer L. Walton-Fisette, Sue Sutherland, Michelle Flemons, Alan Ovens, Sharon Phillips and Sara B. Flory	2018	“This project sought to map variations in definition and conceptualiztion of social justice and sociocultural issues among physical education teacher educators (PETEs) and physical education and sport pedagogy (PESP) educators.”	Qualitative	HQ
42	In defence of white privilege: physical education teachers’ understandings of their work in culturally diverse schools	Dean Barker	2019	“The objective of this investigation was to investigate this claim and generate an understanding of how white PE teachers in a culturally diverse high school make sense of their work with non-white students.”	Qualitative	HQ
43	“Singled out because of skin color … ”: exploring ethnic minority female teachers’ embodiment in physical education	Mara Simon and Laura Azzarito	2019	“Purpose: To explore how race and gender intersect in the lived experiences of ethnic minority female PE teachers in predominantly white schools in the United States.”	Qualitative	HQ
9	“I just treat them all the same, really”: teachers, whiteness and (anti) racism in physical education	Anne Flintoff and Fiona Dowling	2019	This paper explores physical educators perspectives on race and racism as a first step towards disrupting whiteness and supporting the development of antiracist practice.	Qualitative	HQ
44	“Putting Blinders on”: Ethnic Minority Female PE Teachers’ Identity Struggles Negotiating Racialized Discourses	Mara Simon and Laura Azzarito	2019	“The authors examined the embodied identities of ethnic minority female physical education teachers who work in predominantly white schools to identify how whiteness informs their sense of self.”	Qualitative	HQ
4	Remember the titans: The lived curriculum of Black physical education teacher education scholars in the U.S	Langston Clark, Marcus W. Johnson, Latrice Sales and LaGarrett King	2020	“The intent of this article is to present four historical physical education teacher educators as role models for intellectuals in academia and in particular, Black scholars in teacher education”	Conceptual/theoretical	
45	Intersections of Forced Migration and Gender in Physical Education.	Fabienne Bartsch and Bettina Rulofs	2020	“Against this background [stereotypes again student from refugee background], through an interview study with 31 PE teachers from schools in Germany, we investigate teachers’ perspectives on young people from refugee backgrounds. Specifically, we examine which intertwined gendered and racialized patterns of perception and interpretation become apparent when PE teachers talk about this group of students.”	Qualitative	HQ
46	The Emotionality of Whiteness in Physical Education Teacher Education 2020 National Association for Kinesiology in Higher Education Hally Beth Poindexter Young Scholar Address	Mara Simon	2020	“This visual narrative inquiry explored self-identified Black and Latinx pre-service physical education teachers’ (*n* = 10) stories of a racialized identity within predominantly White PETE programs as well as the emotionality of whiteness for the author as a White researcher and teacher educator.”	Qualitative	HQ
22	Structural Discrimination in Physical Education. The “Encounter” Between the (White) Norwegian Teaching Content in Physical Education Lessons and Female Students of Color's Movements and Expressions	Erik Aasland and Gunn Engelsrud	2021	“The Purpose of the Paper is Twofold: (i) To investigate what kind of movements, knowledge/skills and bodies are valued and privileged, or ignored/discriminated in teaching practices in PE. (ii) To depict the “encounter” between the teaching content in physical education lessons and the female students of color's movements and expressions.”	Qualitative	HQ
19	#SayHerName: Black Women Physical Education Teachers of the Year	Tara B. Blackshear	2022	“Drawing on Black feminist thought and critical race theory, the purpose of this article is to describe and explain the experiences of Black women physical educators who earned PE Teacher of the Year.”	Qualitative	HQ
47	Cracks in the narrative: Black and Latinx pre-service PE teachers in predominantly white PETE programs	Mara Simon and Korey Boyd	2023	“This study aimed to understand Black and Latinx pre-service PE teachers negotiations of whiteness, and the accompanying emotional load, at predominantly white institutions (PWIs).”	Qualitative	HQ
48	Meritocracy and material consequences at the intersection of race, class, and disability for pre-service PE teachers	Cory Dixon, Mara Simon and Korey Boyd	2025	“This study explores how Black and Latine pre-service PE teachers at predominantly white institutions (PWIs) face hurdles and/or barriers to teacher licensure, exemplifying material consequences of classism and ableism.”	Qualitative	HQ
49	Basketball shorts, plantation food, and ponytail weaves: Black teachers’ experiences of becoming and being a Physical Education teacher	Louis Francis-Edge, Annette Stride and Hayley Fitzgerald	2025	“This paper explores Black teachers’ lived experiences of becoming and being a PE teacher.”	Qualitative	HQ
50	Teachers’ constructions of female Students of Color as “the other” in physical education	Erik Aasland, Emilie Strand, Ingfrid Mattingsdal Thorjussen and Kristin Walseth	2026	“In order to investigate how institutionalized pedagogical practices produce racialized others, we will examine the narratives of PE teachers about female Students of Color.”	Qualitative	HQ

## 4 Results

Overall, 56 publications met the inclusion criteria, of which 23 focused on PE teachers and PETE students and were included in the present analysis. Among the 23 included publications, four used theoretical, conceptual, or methodological approaches, 17 used qualitative methods, one used a quantitative method, and one followed a mixed-methods approach. All the included publications were published in English, and one originated in Germany. Using CASP and MMAT, each empirical publication was assigned to the highest of the three quality categories. During the TA, four themes were constructed as coherent and meaningful patterns within the data that capture significant aspects of the research topic in relation to the research question. (1) “How racism is understood”: describing and interpreting the underlying concepts of racism in the included publications; (2) “Perspectives of PE Teachers and PETE Students”: pervasive experiences of racism, sexism, and biases among PE teachers and PETE students, emphasizing microaggressions, stereotypes, and a need for structural changes to promote a racially critical pedagogy; (3) “Unveiling Whiteness”: a critical examination of racial hierarchies in physical education and the need for inclusive and counter narratives; (4) “Addressing Gaps in Anti-Racist Training”: emphasizing the need for targeted anti-racist training in PE education to address gaps in the curriculum, raise awareness about whiteness, and tailor interventions to local contexts.

## 5 Main research findings

This section discusses the outcomes and the four constructed themes, focusing on the perspectives of teachers and PETE students. Some of the following quotes include racist content and perspectives. Despite their problematic nature, the goal is to examine them critically, which is a fundamental step in anti-racist efforts to expose and understand racism. This process aligns with the necessity for “simultaneous learning and unlearning of ideological underpinnings that organize dominant social relations” (51). Findings and discussion are integrated throughout. We use “we” or “our” to signal our interpretations.

### 5.1 How racism is understood

Racism is understood through multiple theoretical lenses, creating conceptual diversity that both enriches and complicates understanding. The reviewed publications do not offer a single definition but instead draw on diverse theoretical frameworks that shape distinct conceptualizations of racism. Rather than seeking to define racism in its entirety, the publications often focus on particular dimensions of whiteness or manifestations of racial inequality. This fragmentation becomes particularly significant when examining the concept of whiteness which plays a prominent role in 11 included publications (34, 36–38, 42–47, 50). Whiteness, also conceptualized as a system of privilege, refers to the notion that society is structured hierarchically. In this system, individuals perceived as white have easier access to power than other groups (9). Flintoff and Dowling (9) further elaborate this concept as follows:

“Understanding whiteness as a racial discourse (rather than simply the attributes of white people) it is possible to explore how white cultural practices as promoting a certain racial hierarchy and distribution of embodied capital and rights.”

Understanding whiteness as a discourse rather than an identity creates the foundation for examining how the contrary perspective of viewing the world through the lens of a white-centric society results in the alienation of those who do not “conform”. This idea gives rise to the notion of the “racialized other” which causes an attitude to view others through a “deficit” lens and “lack” of the white “norm” (9). This idea can also be found in ten other articles (22, 35, 37, 38, 43, 44, 47–50).

During the thematic analysis, geographical patterns and the racial positionality of first authors emerged as relevant dimensions of how racism was conceptualized. Among the eight publications from the U.S., two first authors explicitly reported racial or ethnic positionalities that were categorized as PoC in our analysis: Blackshear (19) identified as a Black woman, while Sato described himself as a Japanese native (40). Simon's racial positionality was not explicitly stated in Simon and Azzarito (43), whereas she explicitly identified as white in Simon and Azzarito (44) and explicitly identified as white in subsequent publications (46, 47). In two of these eight publications (4, 43), the racial positionality of the first author was not reported; However, the first authors who self-identified as PoC used different approaches to conceptualizing racism. Sato and Hodge (40) apply the idea of colorblindness, whereas Blackshear (19) refers to Gendered Racism. Across publications authored or co-authored by Simon, racism is explained three times through the concept of whiteness, four times through the concept of The Racialized Other (44, 46–48), twice through Critical Whiteness Studies (46, 47), and once through colorblindness (46); while Francis-Edge et al. (49) examine racism through selected elements of CRT. Other studies published in the UK, New Zealand, Canada, Norway, Spain, and Germany cannot be compared, as the authors either exclusively self-indicated exclusively as white or because there was only one article published within one country. This distribution shows how racism research is concentrated in distinct academic contexts, limiting cross-cultural understanding. Despite Meghji's (52) call for complementary perspectives, this analysis would benefit from a shared conceptualization of racism.

Beyond whiteness, the literature reveals additional intersectional dimensions that complicate traditional racism analysis. The publications address the distinction between binary genders (45), racialized stereotypes (9), gender-specific racism (19, 50) and classism and ableism as other influential forms of discrimination (48). In addition, concepts such as CRT (38, 43, 48, 49), CWS (22, 46, 47, 50), and structural racism were considered (38, 43), followed by the idea of colorblindness (34, 43). The concept of biological racism is mentioned in three publications and utilized to imply how society partly justifies student performance (9, 38, 45).

Given the range of approaches identified, the next step is to situate them within the academic discourses from which these publications originate. Based on the inclusion criteria of this systematic review, all the selected publications are located within the fields of PE and PETE. Furthermore, as previously mentioned, many articles incorporate elements of CRT. Given the prominence of CRT, these sources can be situated within both PE and CRT. In addition to these domains, perspectives from the Sociology of Education (22, 33, 34, 42, 45) and Psychology (39) are also evident. As expected, articles based on CRT tend to focus on structural racism, whiteness, and intersectionality within educational contexts. Contributions grounded in sociological perspectives emphasize institutional norms, privilege, and systemic inequalities—issues which are also central in CRT. Furthermore, the concept of whiteness emerges as a transdisciplinary issue that permeates all disciplinary perspectives. It is not possible to identify a disciplinary field or theoretical lens that adheres to a unified definition of racism. Rather, what emerges is a rich intersection between pedagogical practice and the diverse conceptualizations of racism found across the literature. In sum, racism is perceived through multiple lenses depending on the corresponding research focus, rendering it a complex theme that lends itself to an intersectional approach.

### 5.2 Perspectives of PE teachers and PETE students

The lived experiences of PE teachers and PETE students reveal how racism operates at both interpersonal and institutional levels. Teachers experienced racist encounters in various forms. The following excerpt elucidates their feelings:

“All three participants spoke about the ways in which micro-aggressions, or minute negative interactions, made them feel isolated and frustrated by the lack of diversity within their teaching contexts” [(47), p. 260].

Building on this passage, the literature further reveals that experiences of racial discrimination are not limited to practicing teachers. They are also evident in every publication addressing marginalized PETE students. Their accounts support CRT's view that racism is endemic in predominantly white educational systems. Experiences include stereotypes, exclusion, everyday racism, microaggressions, underrepresentation and colorblindness (9, 19, 22, 33, 34, 36, 39, 42–45, 47, 48). A 2025 study shows that Black and Latine PETE students navigate white spaces, challenge norms, and affirm identity (48). Navigating these spaces requires significant emotional labor and strategic self-presentation, challenges that were not described for white students in the reviewed literature. This emphasis aligns with CRT's core tenet of counter-storytelling, centering marginalized voices in education (24). The following passage further underlines the value of insights obtained from counter-storytelling:

“Moreover, the counter story reflects these educators’ tenacity and determination to overcome these challenges, progress in their careers and make a difference in the lives of the young people they teach. Whilst racism is an enduring aspect in these teachers’ lives, they are well versed at negotiating and navigating various barriers” [(49), p. 347].

Yet institutional responses often sustain exclusion despite evidence of racism. PETE students often lack support to report racism:

“Natacha noted how she frequently felt ‘uncomfortable’ by racialized comments but that she didn’t even know who to report it to: ‘Who would I go to’?” [(43), p. 114].

These gaps in institutional structures are further emphasized when racism and classism shape the reality of PETE students:

“Teacher licensure exams, both in terms of content and cost, were a significant gatekeeper to formally entering the teaching profession. Damien described how the three separate state-required exams cost $90 per attempt […] without additional financial support from his institution” [ (48), p. 342].

CRT's intersectional lens reveals how race, gender, and power converge to shape the marginalization of women of color in white-dominated fields. In this context Douglas and Halas (34) noted that, with few role models, individuals have restricted mentorship opportunities:

“Aboriginal faculty and students can experience the faculty as isolating, alienating and exclusive; with few role models, they have limited opportunities for mentorship” [(34), p. 466].

This lack of representation is not just a demographic gap—it is also a structural issue that reinforces exclusion. This excerpt gives insights into the consequences of underrepresentation for students of color:

“How can you become something you can’t see? He says low numbers of minority teachers send a clear message to minority ethnic kids that positions of authority are not available to them” [(49), p. 343].

In addition to the racialization experienced by PE teachers and PETE students, female-identifying individuals encounter sexism, often intensified at the intersection with race (9, 19, 22, 33, 34, 36, 38, 40–45, 47, 50).

Blackshear (19) criticizes “gendered racism in American culture”. Clark et al. (4) included many icons who have shaped Black history:

“[She] was a womanist and bridge leader because she used her agency to stand in the oppressive gaps created by the overt racism, sexism, and classism of her day” [(4), p. 512].

Turner's legacy reflects ongoing struggles with intersecting oppression in today's education. The discrimination she faced still persists in PE, often maintained (un)consciously by white educators. This was particularly evident in the included publications (9, 19, 22, 42, 45, 47, 50). The following quote underscores how whiteness and gender intersect to shape deficit-based perceptions of female students of color:

“In the eyes of the teachers, the female students of color's movements and bodies are seen as ‘deficient’ and ‘out of place’” [(22), p. 778].

The following excerpt illustrates how female students of color face stereotypes expressed by their PE teachers:

“I’ve noted that these girls frequently don’t bother to get involved. Their lack of response often leads to discouragement. This situation poses challenges for both parties: the girls feel insecure and their peers’ efforts to involve them are met with silence, creating difficulties for everyone involved” [(50), p. 9].

By contrast, a quantitative study from Spain reveals a positive attitude of PETE students towards diversity (39). However, this contrasts with most qualitative findings, which highlight the persistence of unconscious biases among teachers. Furthermore, Flintoff and Dowling elaborate the following:

“Although many of our educators talked about fighting for inclusive practice and equal rights, nevertheless, negative stereotypes about racialized others are woven into the texts […]” [(9), p. 129]

illustrating how the normativity of whiteness despite intentions toward inclusivity, revealing the gap between conscious intentions and unconscious practices. This socially ingrained construct of structural racism is reflected in numerous unconscious stereotypes that explain why PE and its instructors are perceived as colorblind (9, 19, 44, 47, 48). This is consistent with the observation that teachers work within the construct of a hidden curriculum dominated by white privileges, which appears to shape their attitudes. CRT rejects whiteness as a neutral norm, revealing its institutional power (53). Simultaneously, their actions contributed to the perpetuation of this hidden curriculum (9, 41, 44). This assumption is reinforced by Sato and Hodge (40), who indicated that

“some White teachers are fearful of looking inward to examine and share their beliefs and values of hidden conscripts of race, ethnicity, and class” [(40), p. 97].

Although the reviewed publication does not address this, other studies indicate that some educators actively seek training in multicultural and anti-racism education (54).

Therefore, structural change requires more than individual willingness. It demands institutional commitment to supporting critical reflection. We assert that teachers must have the opportunity to reflect on and deconstruct their privileges and (unconscious) biases and subsequently learn to work in a racially critical manner.

### 5.3 Unveiling whiteness

The prevalence of whiteness in PE extends beyond demographic representation to encompass systemic control of knowledge, practices, and institutional culture. Among the 23 publications, 16 discuss the prevalence of whiteness as a system of norms in PE across Western countries (4, 9, 19, 22, 33, 34, 36, 38, 41–48). This body of literature addresses several key dimensions, ranging from the overrepresentation of white individuals in leadership and positions of power to the systematic marginalization of those who are “different” within PE. The reviewed literature questions who controls dominant narratives and stresses grounding research in lived experience. First, most PE educators are identified as white—often in contrast to diverse student populations. Douglas and Halas (34) highlighted that predominantly white settings are often unconsciously established by white individuals and are not officially recognized or intended. However, this environment perpetuates racial inequality by maintaining and reinforcing a social structure in which the racial hierarchy is preserved. Additionally, as Francis-Edge et al. (49) have underscored, the underrepresentation has a direct impact on the students in PE classes, as they lack role models. Second, other publications have predominantly addressed white PETE students. Additional publications predominantly reported on white faculty members in university teaching positions. These findings highlight the structural dominance of whiteness across PETE programs, reinforcing CRT's assertion that white normativity permeates educational leadership (4, 9, 19, 22, 33, 34, 36, 38, 41–50).

Furthermore, Flintoff highlights that the available data indicate that young white men predominantly occupy leadership roles (36). In addition to these person-related observations, two publications discussed white ideas in teaching:

“The teachers’ embodiment of a white habitus (Bonilla-Silva et al., 2006) and its sense of superiority and desire to educate non-white students into white ways of doing things” [ (42), p. 143].

Bartsch aligns with this idea and describes similar attitudes of teachers, stating the following:

“This colonial idea of controlling, cultivating, and disciplining the body of the ‘other’ that lies outside the West through sports and PE can also be detected” [ (45), p. 18].

These examples show how “neutral” practices uphold cultural dominance. Therefore, PE—intertwined with the world of sports—has been criticized for marginalizing those deemed “different”, typically defined in terms of a norm associated with whiteness, masculinity, heterosexuality, and able-bodiedness (38). Many studies apply an intersectional lens in their analysis (4, 19, 33, 38, 39, 45, 48, 50). Additionally, racialized minority PETE students and teachers may perceive the environment as isolating, alienating, and exclusive, resulting in limited access to role models and mentorship. Furthermore, the overrepresentation of white individuals creates a feedback loop that reinforces exclusion while limiting learning opportunities for all participants. This restriction ultimately deteriorates the educational experience for everyone by limiting exposure to diverse perspectives, teaching methods, and cultural knowledge that could enrich PE practice. Thus, it is crucial to engage with the topic and articulate the following:

“One important consequence is that the degree program sustains the racial hierarchies that reproduce and maintain the prevailing (white) cultural character of institutions of higher education” [(34), p. 470].

This structural critique is embodied in the legacy of Anita J. Turner, whose commitment to social justice significantly benefited Black Americans and, more broadly, society as a whole (4). Such efforts parallel anti-racist initiatives that interrogate whiteness—not as a loss for white individuals, but as an opportunity for self-awareness and shared progress. This reflective process fosters a deeper understanding of privilege and is essential for achieving a more inclusive society (55). Crucially, creating an inclusive society also means empowering people of color to tell their own stories. As Blackshear (19) warns:

“Black women's narratives, especially in PE, are narrated and controlled by non-Black scholars who lack the embodied experiences of Black women.”

In addition, this publication is also shaped by whiteness, as the first and last authors identify as white men. In 15 of the 23 included publications, the racial positionality of the first author was addressed or reported, while in eight publications it was not. Among those reporting their positionality, ten first authors identified as white (9, 22, 33, 36, 42, 44–47, 50). It can be reasonably inferred that most of the remaining authors are also predominantly white scholars.

This raises fundamental questions about knowledge production and representation in racism research. We must critically examine why white scholars—rather than those who share the participants’ backgrounds—possess the access, power and resources to narrate the experiences of marginalized students. This concern aligns with CRT's critique of dominant knowledge production and its emphasis on epistemic injustice, particularly in academic spaces where marginalized voices are often excluded or mediated by those in positions of privilege (24). This critique extends to our own work, highlighting the ongoing challenge of conducting anti-racist research within white-dominated academic structures. The special issue on Black Scholarship in Physical Education highlights the need for more equitable authorship and greater representation of Black scholars in shaping discourse within PE research (56). Some articles from this special issue are also featured among the publications reviewed here.

Additionally, Dei (57) advocates that research and policy be grounded in communities that are most affected by the generation of knowledge in research and policy decisions.

In summary, a critical initial move towards an anti-racist approach in PE and PETE involves examining the dynamics of whiteness and shifting focus from the Racialized Other to whiteness itself (9). The first step in the employment of whiteness as an analytical framework is to understand our roles and experiences as teacher educators and teachers in a critical and reflexive manner (58). Ideas for achieving this can be found in the following theme: “Addressing Gaps in Anti-Racist Training”.

### 5.4 Addressing gaps in anti-racist training

The previous themes reveal systemic problems that demand structural solutions through comprehensive anti-racist education. We focused on the needs and possibilities for PETE students and teachers. Several publications have criticized the absence of a

“specific course on race in the overall curriculum” [(34), p. 469]

for PETE. This absence extends to courses that prepare teachers or PETE students to effectively engage with and support multicultural and multiethnic classes (36, 39, 43). Furthermore, the need identified in the previous theme “Unveiling Whiteness” is that white PE teachers and PETE students should become aware of their whiteness. Therefore, it is crucial to address whiteness, which is often associated with dominant constructions of masculinity, as an essential step towards unraveling personal biases and privileges. This process enables individuals to leverage their privilege not only for personal advantage but for the benefit of all (59). We conclude that the absence of widespread anti-racist training reflects the institutional resistance to confronting whiteness and racial privilege—a key critique of CRT. Recent research reinforces the urgency of addressing these structural gaps. Dixon et al. (48) highlight how limited structural awareness in PETE marginalizes students affected by race, class, and disability, underscoring the need for social justice–oriented reform. However, two studies also focused on the evaluation of pilot projects (32, 37). In 1990, participants reported that a two-semester class helped them reflect on their point of view and disrupt their way of thinking, demonstrating the potential for structured intervention to create critical consciousness. This becomes apparent in the following quote from the teacher:

“I have become aware that I am racist to some extent. I once thought that I was not and now realize my own prejudices” [(32), p. 122].

In a course designed for PETE students, the authors integrated instructional sessions for knowledge acquisition with practical observations and teaching sequences in a multiethnic school setting. The course was complemented with a self-reflective learning journal (61). The aforementioned element was also incorporated into a six-week intervention involving service-learning within a diverse multiethnic school context described by Bruce (37). In both publications, the authors discuss not only the challenges but also how students learn to deconstruct their stereotypes. The following was noted:

“there is no evidence to suggest that this short course has had any lasting impact” [(37), p. 242]. Williamson and Williams (32) expand their reasoning by arguing that

“A single semester experience, of course, will not overcome a lifetime of socialized beliefs and attitudes toward racism, sexism, classism, motor elitism, and heterosexism” [(32), p. 121].

Considering the results of the last three themes as well as this one, we conclude that further research and pilot projects on the anti-racist education of teachers and PETE students are needed. As the global phenomenon of racism varies locally (10), it is crucial to learn from each other as well as integrate local differences into program design. While culturally relevant pedagogies were not explicitly discussed in the reviewed studies, it is important to acknowledge that such approaches may incorporate anti-racist content without explicitly identifying it as such. For instance, in the German context, an established and empirically grounded discourse on intercultural education in sports provides a pertinent example (60). The authors featured in the included publications also address this topic in another publication and call for the implementation of Culturally Relevant and Sustaining Pedagogies (CRSP) in PETE education (61). In addition, from our perspective, the starting point should be to understand whiteness as a social construct, along with an examination of one's own biography, privileges, and biases.

## 6 Limitations

In our systematic literature review, we focused on specific keyword combinations and selected databases and excluded studies that did not explicitly address racism. The review was limited to publications in English and German. While this decision was thoughtfully made, it is possible that relevant and valuable publications in other languages may have been overlooked. Themes were initially developed from a white male perspective, emphasizing the need to consider individual biases in shaping topics. To minimize this effect, the work was critically reflected by the other authors. A key decision in our study was to include perspectives from a field outside the faculty. The review mainly identified qualitative studies in English-speaking countries, revealing a scarcity of large-scale quantitative surveys on racism-critical physical education from teachers' perspectives. Among publications reporting first-author racial positionality, white first authors predominated, while eight publications did not report first-author racial positionality. This pattern underscores Dei's (57) call for research grounded in communities directly affected by knowledge generation. We are aware that the absence of non-white authorship reflects the power dynamics CRT critiques—where dominant groups control knowledge production and shape discourse about race.

## 7 Conclusion

Our review identified promising high-quality international literature, predominantly employing qualitative approaches. This study explores the complexity of defining racism by presenting 23 included publications with authors employing diverse concepts such as whiteness studies, racialized otherness, CRT, and colorblindness. The sum of all publications highlights the complexity of understanding racism across different societal contexts. The analysis of the perspectives of PE teachers and PETE students reveals pervasive experiences of racism and discrimination within educational settings. Microaggressions, stereotypes and systemic biases contribute to an environment that marginalizes individuals based on race and gender. Analysis of 23 publications shows whiteness as a dominant theme, with white individuals overrepresented as educators, students and faculty. This unequal distribution reinforces the perception of white people as racially unmarked while the “others” are marginalized, highlighting the need for more inclusive perspectives within the field. Institutions and journals should support scholars from racially minoritized groups through inclusive funding, collaboration, and mentorship initiatives. This study emphasizes the need for anti-racist education, particularly addressing gaps in training for PE teachers and PETE students. Teacher training must equip (especially white) educators to examine bias, reflect on their racial identity, and challenge norms of whiteness.

As racism manifests globally but exhibits local variations (10), the analysis underscores the necessity for global solution strategies. These strategies require local adaptation in practice and academia. A starting point could involve the examination and critical reflection of predominantly white- and male-dominated domains by employing approaches such as CWS. Global findings must be aligned with local needs to design targeted professional development.

Future research should examine how institutional racism and white epistemologies restrict who produces race research and whose voices are legitimized. Scholars must also reflect critically on their work to avoid overburdening researchers of color.

## Data Availability

The original contributions presented in the study are included in the article/Supplementary Material, further inquiries can be directed to the corresponding author.
